# Sigmoid model analysis of breast dynamic contrast‐enhanced MRI: Distinguishing between benign and malignant breast masses and breast cancer subtype prediction

**DOI:** 10.1002/acm2.13651

**Published:** 2022-05-20

**Authors:** Norikazu Koori, Tosiaki Miyati, Naoki Ohno, Hiroko Kawashima, Hiroko Nishikawa

**Affiliations:** ^1^ Department of Radiology Komaki City Hospital Komaki Aichi Japan; ^2^ Division of Health Sciences Kanazawa University Graduate School of Medical Sciences Kanazawa Ishikawa Japan

**Keywords:** breast dynamic contrast‐enhanced MRI, DCE‐MRI, invasive ductal carcinoma, sigmoid model

## Abstract

Dynamic contrast‐enhanced magnetic resonance imaging (DCE‐MRI) is performed to distinguish between benign and malignant lesions by evaluating the changes in signal intensity of the acquired image (kinetic curve). This study aimed to verify whether the existing breast DCE‐MRI analyzed by the sigmoid model can accurately distinguish between benign and invasive ductal carcinoma (IDC) and predict the subtype.

A total of 154 patients who underwent breast MRI for detailed breast mass examinations were included in this study (38 with benign masses and 116 with IDC. The sigmoid model involved the acquisition of images at seven timepoints in 1‐min intervals to determine the change in signal intensity before and after contrast injection. From this curve, the magnitude of the increase in signal intensity in the early phase, the time to reach the maximum increase, and the slopes in the early and late phases were calculated. The Mann–Whitney *U*‐test was used for the statistical analysis.

The IDC group exhibited a significantly larger and faster signal increase in the early phase and a significantly smaller rate of increase in the late phase than the benign group (*P* < 0.001). The luminal A‐like group demonstrated a significantly longer time to reach the maximum signal increase rate than other IDC subtypes (*P* < 0.05).

The sigmoid model analysis of breast DCE‐MRI can distinguish between benign lesions and IDC and may also help in predicting luminal A‐like breast cancer.

## 1 INTRODUCTION

In breast cancer diagnosis using magnetic resonance imaging (MRI), dynamic contrast‐enhanced MRI (DCE‐MRI) is performed for distinguishing between benign and malignant lesions by evaluating the changes in the signal intensity (SI) of the acquired images (kinetic curve). Kuhl et al. reported that if the SI in the late phase is lower than that in the early phase of the kinetic curve contrast in breast tumors, the probability of malignancy is high.^1^ Furthermore, this method has been employed in the Breast Imaging Reporting and Data System (BI‐RADS) MRI.^2^

Recently, subtype classification has been used to determine the most appropriate treatment for patients with breast cancer as an alternative to the genetic testing currently performed, which is quite expensive. Alternative subtype classification is performed based on the presence or absence of estrogen receptors (ER), progesterone receptors (PgRs), and human epidermal growth factor receptors 2 (HER2) in biopsy samples.

Previous studies have demonstrated that the subtype classification is also possible using the tumor morphology information obtained by MRI with diffusion and quantitative analyses from DCE‐MRI.^3^, ^4^, ^5^, ^6^, ^7^, ^8^, ^9^ Electrical properties tomography and magnetization transfer imaging techniques have been used to improve the diagnosis of benign and malignant breast tumors.^10^, ^11^

However, since these methods involve specialized imaging techniques, the acquisition of images is limited by the capability of the MRI apparatus. In this study, we focused on the sigmoid model,^12^, ^13^, ^14^ which can evaluate the dynamic characteristics of contrast media through the analysis of existing DCE‐MRI images.

The purpose of this study was to evaluate the applicability of this sigmoid model in differentiating benign from invasive ductal carcinoma (IDC) and in predicting tumor subtypes.

## 2 METHODS

### 2.1 Participants

The study was a single‐center, non‐interventional, retrospective study. This study was approved by the Institutional Review Boards of the participating institutions, and informed consent to participate was obtained from all patients.

The study included 154 patients (116 with IDC and 38 with benign masses; median age: 53 and 45 years, respectively) who underwent breast MRI for the detailed examination of breast masses in our hospital between April 2, 2017, and April 26, 2019. The patients were selected using a consecutive sampling method. All the patients with malignant lesions underwent histological examinations and were found to have IDC. Benign lesions were histologically or cytologically diagnosed. The IDC subtype was luminal A‐like breast cancer in 71 cases, luminal B‐like breast cancer in 22 cases, HER2 overexpression breast cancer in eight cases, and triple‐negative breast cancer in 15 cases. The data pertaining to patient age, tumor size, and tumor subtype are summarized in Table 1.

**TABLE 1:** Patient and lesion characteristics

	IDC** (*n* = 116)	Benign (*n* = 38)	*P* value
Age (years)*	53 (29–84)	45 (22–84)	<0.01
Diameter (mm)*	17 (5–67)	10 (5–32)	<0.001

Histologic type, *n*/total *n* (%)	
Luminal A	71/116 (61.2%)
Luminal B	22/116 (19.0%)
HER2***	8/116 (6.9%)
TN****	15/116 (12.9%)

*Notes*: *Data are the medians, and numbers in parentheses are the ranges.

** IDC:Invasive ductal carcinoma.

*** HER2: HER2 overexpression.

**** TN: Triple‐negative breast cancer.

In addition, 29 cases with normal tissue samples were judged to have benign diseases, five cases were classified as fibroadenoma, two cases were diagnosed as intraductal papilloma, and there was one case each of fibrocystic disease and sclerosing lesion, accounting for a total of 38 cases. The pathological diagnoses of IDC were based on surgical specimens in 110 cases, and the histological diagnoses were determined via needle biopsy in six cases. One case of benign disease involved a pathological diagnosis based on a surgical specimen, 18 cases involved histological diagnoses via needle biopsy, and 19 cases involved cytopathological diagnoses via puncture suction biopsy.

### 2.2 Imaging method

Imaging was performed with a 1.5‐T magnetic resonance system (Signa Excite HDxt Horizon, GE Healthcare, Waukesha, USA) and an 8‐channel breast coil. Images were collected at a total of seven timepoints by volume imaging for breast assessment (VIBRANT) using a three‐dimensional T_1_‐weighted gradient‐echo sequence combined with fat suppression, before the injection of the contrast medium and then every minute from 30 s following the start of the injection. Further, the left and right mammary glands were individually shimmed to suppress the fat more evenly. The VIBRANT sequence protocol was as follows: echo time = 2.2 ms; repetition time = 4.5 ms; flip angle = 10°; slice thickness = 2.0 mm; field of view = 320 × 288 mm; in‐plane resolution = 1.0 × 1.25 mm; average number of samples = 1; bandwidth = ± 62.5 kHz; and reduction factor = 1.75. The fat suppression method was the spectral pre‐saturation with inversion recovery technique, and the imaging time was 66 s. The contrast agent used was gadobutrol (Gadovist; Bayer Pharma AG, Leverkusen, Germany), a gadolinium‐based contrast agent for MRI. It was intravenously administered as a bolus at a rate of 1 ml/s (0.1 mmoL/kg) through a hand dorsal vein line (20 G), which was flushed with 40 ml of saline solution through a power injector (Sonic Shot GX, Nemoto Kyorindo, Tokyo, Japan).

### 2.3 Histopathological diagnostic method

The histopathological analyses were performed by a pathologist. For malignant lesions, the presence or absence of ER and PgR was determined by immunohistochemistry; HER2 was identified based on the ASCO/CAP HER2 inspection guidelines.^15^ The luminal A‐like subtype was defined as tumor cells positive for ER and/or PgR, negative for HER2, and < 20% for MIB‐1; the remaining malignant subtypes were classified as other IDC groups. Benign lesions were included in a different group.

### 2.4 Analysis procedure

**FIGURE 1:**
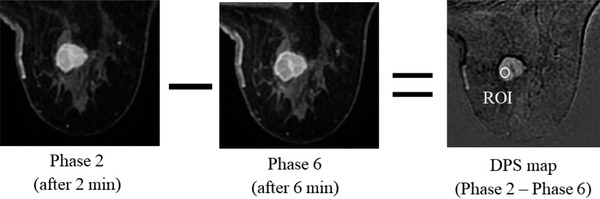
An example of region‐of‐interest (ROI) placement using a dynamic phase subtraction map (DPS‐map). The DPS‐map is based on the subtracted image between phase 2 and phase 6 after a contrast agent was administered. The DPS map is from a lesion located in the right breast of a 52‐year‐old female patient diagnosed with invasive ductal carcinoma (IDC).

The region of interest (ROI) placements and image analysis were performed by one radiological technologist with 10 years of experience in MRI. ROIs were set only for recognizable mass lesions ≥ 10 mm^2^, using a circle ≥ 5 mm^2^ in area. The signal was enhanced based on the dynamic phase subtraction (DPS) map,^16^ and the ROI was placed in the mass region, while excluding the feeding blood vessel. Basically, we used a single ROI in each phase, but when the position of the lesion fluctuated due to patient movement or other factors, we manually reselected the ROI (Figure 1).

**FIGURE 2:**
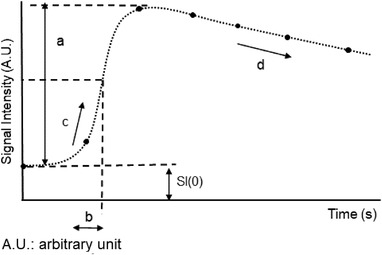
Parameters involved in sigmoid model analysis, where *t* is the time, *a* is the change in signal intensity associated with the contrast medium, *b* is the time to reach the maximum slope of the signal intensity curve, *c* is the slope of the signal intensity curve in the early phase, *d* is the slope of the signal intensity curve in the late phase, and SI (0) is the signal intensity before contrast enhancement.

**FIGURE 3:**
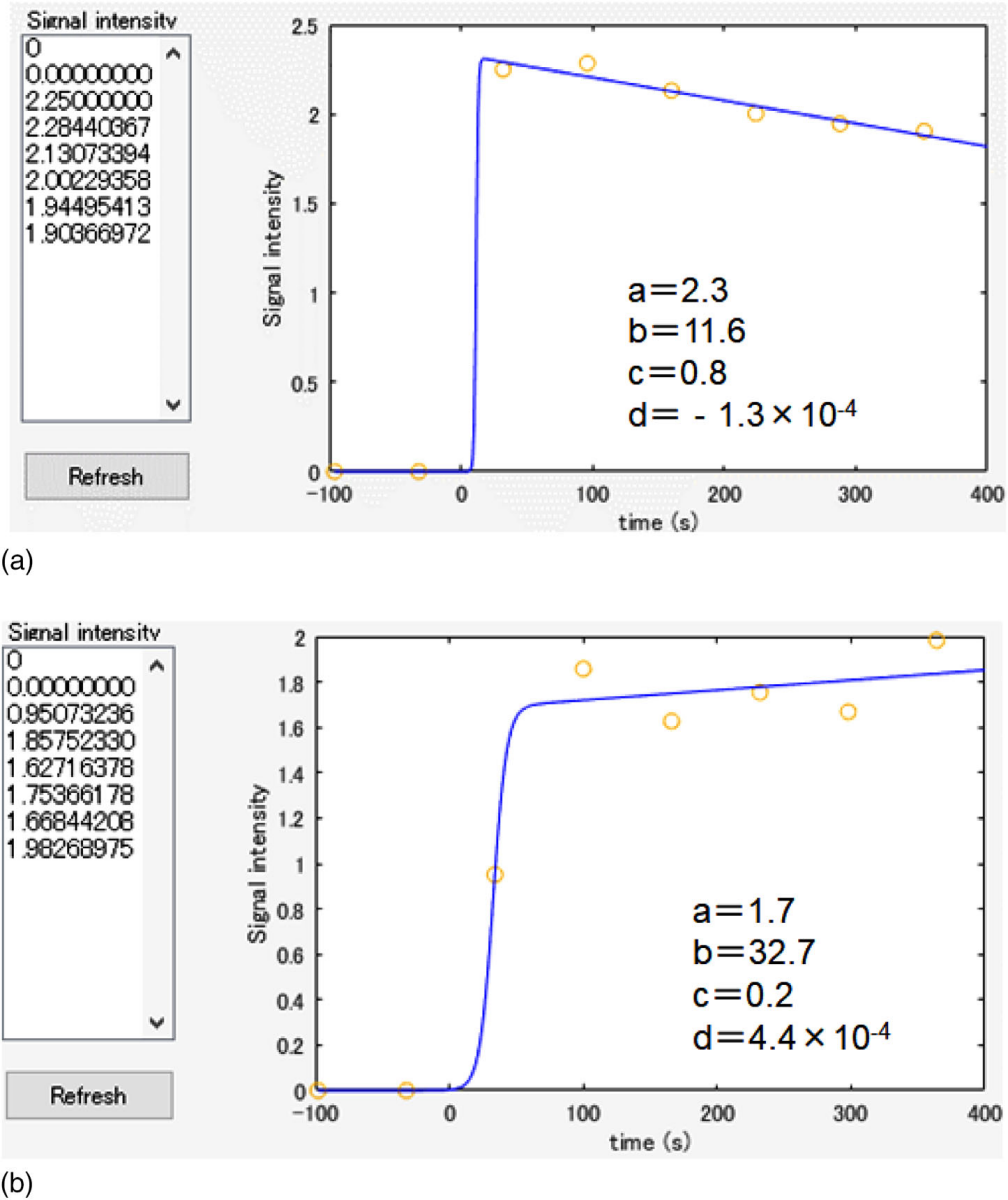
(a) Examples of IDC fitting with sigmoidal models. (b) Examples of benign lesion fitting with sigmoidal models.

An example of the ROI placement using the DPS map is shown in Figure 1. ROIs were set for the seven phase images obtained as described above, and the average SI was measured. The average SI of the ROI for each time‐phase image was subsequently fitted using the nonlinear least‐square fitting method and the Marquadt–Levenberg algorithm^17^ using the sigmoid model, described as follows:

(1) $$\mathrm{SI}\left(t\right)=\frac{a+\left(d\cdot t\right)}{1+\exp\left(-\frac{t-b}{c}\right)}+\mathrm{SI}\left(0\right),$$

where *t* is the time, *a* is the change in SI due to the contrast medium, *b* is the time until the SI curve reaches the maximum slope, *c* is the slope of the SI curve in the early phase, *d* is the slope of the SI curve in the late phase, and SI (0) is the signal intensity before contrast enhancement (Figure 2). The graph of the resulting model is shown in Figure 2. Based on this methodology, MATLAB software (Mathworks, Natick, MA, USA) was created to conduct the analysis. Examples of the sigmoidal model fitting for IDC and benign lesions are shown in Figure 3a,b.

Equations (2) and (3) show the initial phase enhancement rate and delayed phase enhancement rate used in BI‐RADS.

(2) $$\mathrm{Initial}\;\mathrm{phase}\;\mathrm{enhancement}\;\mathrm{rate}=SI_{\mathrm{ph}1}/SI_{\mathrm{pre}},$$

(3) $$\mathrm{Delayed}\;\mathrm{phase}\;\mathrm{enhancement}\;\mathrm{rate}=SI_{\mathrm{ph}2}/SI_{\mathrm{ph}6},$$

where SI _ph1_ is the signal intensity of contrast enhancement phase 1, SI_pre_ is the signal intensity of the pre‐contrast phase, SI _ph2_ is the signal intensity of contrast enhancement phase 2, and SI _ph6_ is the signal intensity of contrast enhancement phase 6.

### 2.5 Statistical analysis

The Mann–Whitney *U*‐test was used for statistical analysis. Each fitting parameter obtained using the above‐mentioned procedure was compared between the benign and IDC groups and between the luminal A‐like subtype and the other IDCs. Receiver operating characteristic (ROC) curves were constructed to demonstrate the accuracy of assessing the differences in the various parameters between the IDC and benign groups. R software (version 3.4.1, R Foundation, Vienna, Austria) was used for the statistical analysis. A *P*‐value of < 0.05 was considered statistically significant.

## 3 RESULTS

Figure 3 shows examples of the fitting of the sigmoid model for IDC and benign lesions.

Table 2 shows the differences in the various parameters between the IDC and benign groups. All the parameters showed a significant difference (Table 2).

**TABLE 2:** Differences in the various parameters between invasive ductal carcinoma and benign masses

Parameter	IDC*	Benign	*P* value	Sensitivity (%)	Specificity (%)	AUC**
Sigmoid model analysis						
*a*	1.5 ± 0.4	1.4 ± 0.8	<0.01	42.1	92.1	0.64 (95% CI: 0.51–0.76)***
*b* (s)	19.6 ± 8.2	31.5 ± 13.5	<0.001	84.2	67.5	0.83 (95% CI: 0.76–0.90)
*c*	0.3 ± 0.3	0.1 ± 0.1	<0.001	50.0	91.2	0.72 (95% CI: 0.62–0.82)
*d* [×10^−4^]	−3.7 ± 9.6	2.2 ± 19.9	<0.001	86.8		0.70 (95% CI: 0.60–0.80)

Kinetic post‐contrast curve						
Initial phase enhancement ratio (%)	121.1 ± 39.8	79.5 ± 47.5	<0.001	71.1	87.9	0.79 (95% CI: 0.69–0.89)
Delayed phase enhancement ratio (%)	98.0 ± 11.8	107.9 ± 18.4	<0.001	84.2	55.2	0.72 (95% CI: 0.62–0.82)

*Notes*: *IDC: invasive ductal carcinoma.

** AUC: area under the curve.

*** 95% CI: 95% confidence interval.

**FIGURE 4:**
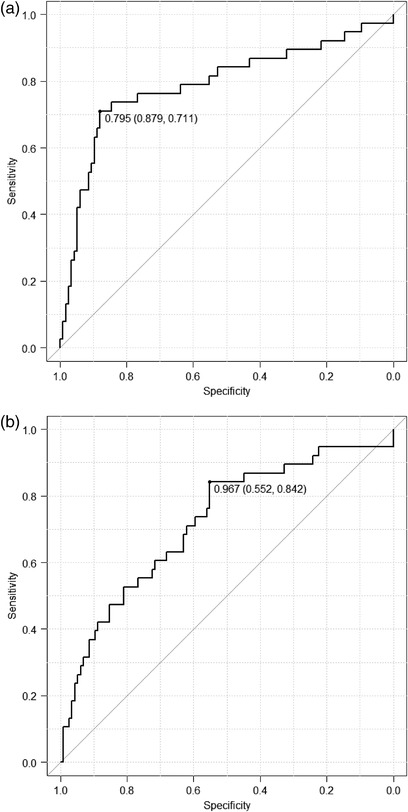
(a) Area under the receiver operating characteristic curve of the initial phase enhancement rate. The area under the curve is 0.79 (95% confidence interval, 0.69–0.89). When the threshold was 0.795, the sensitivity and specificity were 71.1% and 87.9%, respectively. (b) Area under the receiver operating characteristic curve of the delayed phase enhancement rate. The area under the curve is 0.72 (95% confidence interval, 0.62–0.82). When the threshold was 0.967, the sensitivity and specificity were 84.2% and 55.2%, respectively.

**FIGURE 5:**
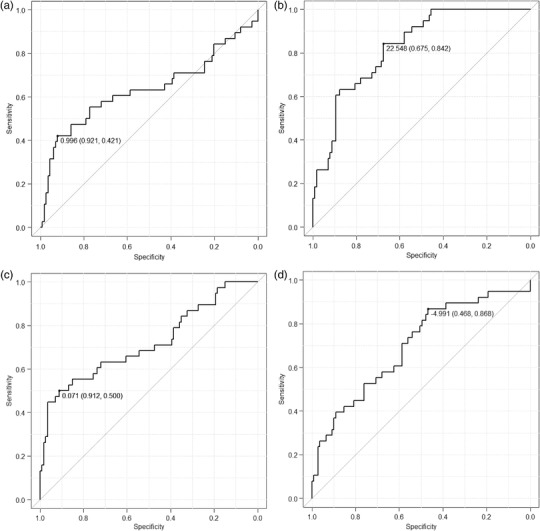
(a) Area under the receiver operating characteristic curve of parameter “*a*.” The area under the curve is 0.63 (95% confidence interval, 0.51–0.76). When the threshold was 0.996, the sensitivity and specificity were 42.1% and 92.1%, respectively. (b) Area under the receiver operating characteristic curve of parameter “*b*.” The area under the curve is 0.83 (95% confidence interval 0.76–0.80). When the threshold was 22.548, the sensitivity and specificity were 84.2% and 67.5%, respectively. (c) Area under the ROC curve of parameter “*c*.” The area under the curve is 0.72 (95% confidence interval 0.61–0.82). When the threshold was 0.071, the sensitivity and specificity were 50.0% and 91.2%, respectively. (d) Area under the receiver operating characteristic curve of parameter “*d*.” The area under the curve is 0.70 (95% confidence interval, 0.60–0.89). When the threshold was –4.991, the sensitivity and specificity were 86.8% and 46.8%, respectively.

Figure 4, 5, 6 show the results of the ROC curve analysis for each parameter as well as the amount of signal change at the Initial phase enhancement ratio and Delayed phase enhancement ratio of the conventional BI‐RADS.

**FIGURE 6:**
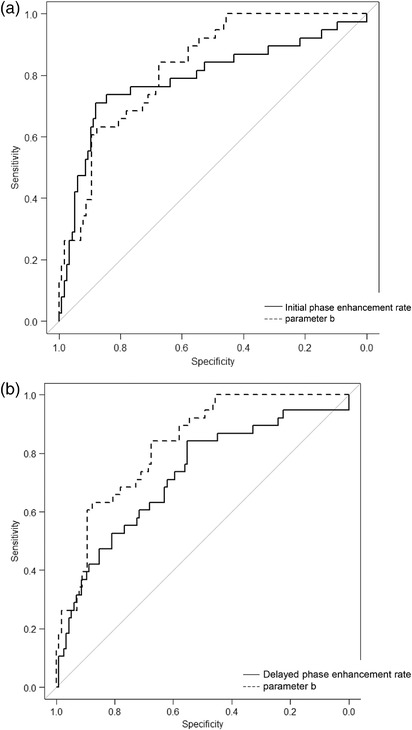
(a) Comparison of the area under the receiver operating characteristic curve of the initial phase enhancement rate and parameter “*b*.” The areas under the receiver operating characteristic curve for the initial phase enhancement rate and parameter “*b*” are 0.79 and 0.83, respectively, with no significant difference (*P* = 0.46). (b) Comparison of the area under the receiver operating characteristic curve of the delayed phase enhancement rate and parameter “*b*.” The areas under the receiver operating characteristic curve for the delayed phase enhancement rate and parameter “*b*” are 0.73 and 0.83, respectively, with a statistically significant difference (*P* < 0.05).

The area under the ROC curves (AUCs) of the initial phase enhancement rate, delayed phase enhancement rate, and parameters *a*, *b*, *c*, and *d* were 0.79, 0.72, 0.64, 0.83, 0.72, and 0.70, respectively. There was a significant difference between the AUC for the delayed phase enhancement rate and parameter *b* (*P* < 0.05).

Table 3 shows the differences in the various parameters between the luminal A‐like subtype and the other IDCs groups. The time until the increasing SI curve reached the parameter *b* showed a significant difference (*P* < 0.05).

**TABLE 3:** Differences in the various parameters between the luminal A‐like subtype and the other IDCs groups

Parameter	Luminal A	Other	*P value*
*a*	1.5 ± 0.5	1.5 ± 0.4	n.s
*b* (s)	20.0 ± 13.9	17.8 ± 7.0	<0.05
*c*	0.3 ± 0.6	0.3 ± 0.6	n.s.
*d* [× 10^−4^]	−3.3 ± 10.2	−5.4 ± 10.2	n.s.

n.s.: not significant

## 4 DISCUSSION

The IDC group showed a larger signal increase in the early phase and a shorter time until the maximum rate of signal increase in the early phase than the benign group. The malignant lesions also showed a steeper increase in the signal in the early phase. These findings indicate that a large amount of contrast agent penetrated the IDC lesions in a short period. In contrast, in the late phase, the rate of signal increase was significantly lower in the IDC group than in the benign group. Therefore, the IDC group had faster cell proliferation and contrast agent excretion than the benign group,^1^ consistent with reports of a high maximum slope when using ultrafast dynamic contrast‐enhanced techniques.^18^

The luminal A‐like breast cancer group demonstrated a significantly longer time to reach the maximum rate of signal increase than the other IDC subtypes. In contrast, there was no significant difference in the increase in SI or the maximum slope, indicating a longer time for contrast absorption, despite similar inflow dynamics. Therefore, luminal A‐like breast cancer seemingly has a longer average transit time and a lower blood flow, even in the presence of similar blood volume. This finding is consistent with that a previous study, which reported that *K*_trans_ was significantly smaller in the luminal A‐like breast cancer type.^19^

Luminal A‐like breast cancer is reportedly less malignant than the other IDC subtypes^20^; therefore, the method used in this study may help determine the most appropriate treatment for the patient if differentiation through imaging is possible.

The SI ratio between the early phase (approximately 1–2 min after contrast medium injection) and late phase (approximately 6 min after contrast medium injection) is used to distinguish between benign and malignant masses, according to the current BI‐RADS guidelines.^2^ However, in clinical practice, cases have been observed in which the SI peaks at 3 or 4 min after contrast administration, followed by a signal decrease (washout). In such cases, it can be difficult to determine which BI‐RADS classification should be adopted. This problem could potentially be solved using our sigmoid model analysis method. Moreover, this method is highly versatile since it can be used for images obtained from commonly used protocols. This study demonstrated that this analysis is clinically useful by evaluating the SI in MRI images, allowing the collection of information that is currently only available via biopsy.

The combination of DCE‐MRI and diffusion‐weighted imaging (DWI) is reportedly useful in distinguishing between benign and malignant breast tumors.^21^ However, in our study, the combination with the apparent diffusion coefficient (ADC) value obtained from DWI was not examined. Using the ADC value together with the sigmoid model employed in this study may lead to further improvement in accuracy.

We cannot directly compare the AUC results obtained in the previous studies^9^ because the participants are not exactly the same; however, the fact that the AUCs in our study are either comparable or higher than those reported by Sorace et al. (K^trans^ method using DCE‐MRI) indicates the usefulness of this method.

This study has certain limitations. Only a single, small ROI was used for each lesion, and it might not have captured the heterogeneity of the contrast within the mass. In particular, small lesions, such as ductal carcinoma in situ, may reduce the analytic accuracy.^22^ In addition, although a single contrast agent was used in this study, the shapes of the kinetic curves reportedly differ based on the contrast agent used.^23^ Therefore, using different contrast agents may affect the results. Finally, it was not possible to increase the time resolution to secure the spatial resolution. Hence, it is a matter for future research is to further increase the time resolution. However, the data collection time in the central part of *k*‐space, which determines the image contrast, was short enough, and in fact, b was significantly different between Luminal A and other groups, thus, it was determined that this issue is not a concern in this study.

## 5 CONCLUSION

Sigmoid model analysis of breast DCE‐MRI can distinguish between benign breast lesions and IDC and may also help in identifying luminal A‐like breast cancer.

## CONFLICTS OF INTEREST

The authors declare that there is no conflict of interest that could be perceived as prejudicing the impartiality of the research reported.

## AUTHORS’ CONTRIBUTION

Norikazu Koori: collected the data, conceived and designed the analysis, wrote the paper; Tosiaki Miyati: conceived and designed the analysis; Naoki Ohno: contributed data or analysis tools; Hiroko Kawashima: revising it critically for important intellectual content; Hiroko Nishikawa: revising it critically for important intellectual content.

## Data Availability

Data are available on request from the authors.
